# Smart Data-Driven Optimization of Powered Prosthetic Ankles Using Surface Electromyography

**DOI:** 10.3390/s18082705

**Published:** 2018-08-17

**Authors:** Roozbeh Atri, J. Sebastian Marquez, Connie Leung, Masudur R. Siddiquee, Douglas P. Murphy, Ashraf S. Gorgey, William T. Lovegreen, Ding-Yu Fei, Ou Bai

**Affiliations:** 1 Human Cyber-Physical Systems Laboratory, Florida International University, Miami, FL 33174, USA; jmarq056@fiu.edu (J.S.M.); cleun006@fiu.edu (C.L.); msidd021@fiu.edu (M.R.S.); obai@fiu.edu (O.B.); 2 Department of Veterans Affairs, Hunter Holmes McGuire VA Medical Center, Richmond, VA 23249, USA; douglas.murphy3@va.gov (D.P.M.); ashraf.gorgey@va.gov (A.S.G.); william.lovegreen@va.gov (W.T.L.); 3 Department of Biomedical Engineering, Virginia Commonwealth University, Richmond, VA 23220, USA; fei@vcu.edu

**Keywords:** electromyography, powered prosthetic ankle, parameter tuning, data-driven optimization, Nelder–Mead, Latin Hypercube Sampling

## Abstract

The advent of powered prosthetic ankles provided more balance and optimal energy expenditure to lower amputee gait. However, these types of systems require an extensive setup where the parameters of the ankle, such as the amount of positive power and the stiffness of the ankle, need to be setup. Currently, calibrations are performed by experts, who base the inputs on subjective observations and experience. In this study, a novel evidence-based tuning method was presented using multi-channel electromyogram data from the residual limb, and a model for muscle activity was built. Tuning using this model requires an exhaustive search over all the possible combinations of parameters, leading to computationally inefficient system. Various data-driven optimization methods were investigated and a modified Nelder–Mead algorithm using a Latin Hypercube Sampling method was introduced to tune the powered prosthetic. The results of the modified Nelder–Mead optimization were compared to the Exhaustive search, Genetic Algorithm, and conventional Nelder–Mead method, and the results showed the feasibility of using the presented method, to objectively calibrate the parameters in a time-efficient way using biological evidence.

## 1. Introduction

Recent improvements in prosthetics introduced powered ankles and knees to the amputee gait and the advent of such technologies brought more balance and symmetry to the users. These prosthetic systems are embedded with battery-driven mechanical motors to provide extra torque, and assists the users by propelling them forward without requiring them to supplement excessive metabolic energy. 

Such systems can be controlled and fitted to various user types with different physical features by manipulating the impedance-controlled parameters. The main parameters are the stiffness for the ankle and the amount of power provided by the system [1]. The stiffness parameter controls how the ankle flexes and helps the users to have a smooth transition between various phases in the gait, which makes it crucial to adjust it to fit each user [2,3,4,5]. If the stiffness was set to a low value, it might cause the user to catch himself/herself by consuming more energy in order to prevent falling and, if it were set to a higher value than necessary, the prosthetic gait would introduce more dissidence to the gait and undermine the effectiveness of the system. In addition, the amount of power provided by the leg needs to be adjusted as well, and its value relies on the weight, height, and strength of the user. If this parameter is not set properly, it will result in under-powering or over-powering; under-powering would cause the user to drag the leg, consume more metabolic energy, and have more pressure on the residual limb, while over-powering would provide an excessive push from the system and cause the user to stumble and increase the possibilities of falling. 

The setup for the powered prosthetic ankle (PPA) systems is currently done by certified prosthetists and orthotists (CPO). The CPO tunes the powered system after achieving a good fit for the prosthetic socket and other equipment [6,7,8]. The way the tuning is done is based on the CPO’s observation and the feedback that the user provides. The CPO asks the amputee to walk in an assisted walkway while they change the parameters and observe the gait. The best parameter setup is achieved when the CPO is satisfied with his/her observation. This process is time-consuming as it requires the user to walk back and forth while the CPO manipulates the parameters and observes their gait pattern until they are satisfied with the performance. The current methods are subjective and observation-based, and there are not any tools to evaluate and quantify the fitness of the parameters. In this study, a smart data-driven tuning method was introduced. The proposed method uses physiological data to build an evidence-based method to calibrate the parameters of the PPA system. 

The results of this paper showed the feasibility of using smart search for objectively tuning the powered prosthetic systems in a much shorter time with surface electromyogram (EMG) signals from residual limb. The results of the study found the optimal parameter setup with a far fewer number of iterations comparted to the exhaustive searching. In addition, the results showed that by using this method, it is possible to replace the observation-based tuning, which requires trial and error for tuning with evidence-based smart searching, which uses biological feedback from the amputee while the PPA is in operation.

The rest of the paper is organized as follows. In Section 2, a review of previous works on PPA tuning and optimization techniques are presented. In Section 3, the materials and methods including the sensor system and the processing and optimization are introduced. In Section 4, the results of the implementation of the proposed method are presented. Finally, the results of the study are discussed in Section 5 and the conclusion is presented in Section 6. 

## 2. Related Works

Earlier ideas for active prosthetic legs were developed in 1991 by Popovic et al. [9]. Since then, several prostheses with active power have been developed [10,11,12,13,14,15]. Early researches focused on automating the amputee gait and their results led to the development of quasi-passive ankle foot prosthetics [16,17,18]. Holmberg from Halmstad University [12] introduced a prosthetic ankle that automatically adjusted according to the ground angle, using accelerometer data. Later, Bedard, from Halmstad University as well, ref. [10] made an actuated leg prosthesis for above-knee amputees. Goldfarb and their group from Vanderbilt University Nashville [14] designed and controlled a pneumatically actuated transtibial (TT) prosthesis with powered knee and ankle joints. Huang and their group from the University of Rhode Island Kingston [13] prototyped a smart prosthetic using a redundant actuator concept, which enabled the system to partially function when the prosthesis loses power. In 2011 a breakthrough was made by Grabowski and Herr from the Massachusetts Institute of Technology and Center for Restorative and Regenerative Medicine [11], where they developed a bionic prosthesis that emulates the function of the biological ankle during level walking. The bionic prosthesis provided a net positive work on a range of walking speeds and they showed the improvement in metabolic energy expenditure and the biomechanical pattern of amputee gait.

Recently, several studies have focused on systems to quantify the observation and do the tuning automatically using mechanical sensors. Wang et al. [19] developed a fuzzy logic-based system using the knee angle and phase duration, which can tune the control impedance for a powered knee prosthesis, resulting in increased symmetry for the non-amputee subjects. Huang et al. [20] also used a powered knee with a passive ankle and developed a fuzzy-based cyber expert system using temporal symmetry, stance width, and trunk sway. Wen et al. [21] also worked on powered knee prosthesis and proposed two automatic tuning strategies—parallel and sequential. The system was tested on able-bodied subjects and the results showed the possibility of using such technology to match the ideal knee profile to improve gait. All of the works in this field focused on prosthetic knees and did not consider the powered prosthetic ankles that transtibial amputees operate. In addition, the focus of these works was on mechanical sensors and they did not consider the biological aspect of the gait. Recent studies have shown the possibility of using EMG signals to control exoskeletons and foot orthosis [22,23,24]. Since mechanical sensors show the effect of movement after the movement was made, it was assumed that considering the biological data would provide a physiology-based model that is more robust to control and optimize the prosthetic systems. The assumption was made based on the fact that the motor movement is the result of physiological stimulation in the muscles. Our recent work focused on this assumption by using EMG signals to model the amputees’ gait in a physiological sense, and investigated the sensitivity of the different muscle groups to the changes in the natural gait, and the results showed that the users rely more on the sound leg to complete the gait [25]. Later, a pilot study was conducted to tune a powered prosthetic ankle using multi-channel EMG signals, using the grids of various possible combinations, and the results were compared to the experts’ tuning, showing the possibility of using evidence based on physiology for automatic tuning [26]. It is hypothesized that the use of EMG signals would provide a direct observation from the amputee gait itself and can be used to model the muscular activity, whereas the mechanical sensors are unable to provide direct information about energy expenditure. 

However, the proposed method relied on the data collection from a limited combination of parameters, where a number of possible combinations were collected offline and a grid of stiffness and power parameters was presented. As each parameter can vary from 0 to 100 percent, there are many possible combinations, but only a limited number of parameter combinations were considered. This limitation also constrained the resolution of the search for each parameter. To have a higher resolution, a smaller-sized grid made up of more data is required, which would be highly time-consuming and not practical. The purpose of this study was to explore the data-driven optimization methods to achieve a smart tuning in a short time, using the physiological data acquired from wireless EMG sensors. This study investigated the use of heuristic and numerical optimization methods to explore a better approach in order to optimize the parameter setup for PPA and to converge to the optimal parameter combination without having to go through the exhaustive search. As the state-of-the-art is lacking a model for amputee gait using EMG signals, a data-driven optimization approach was introduced so as to be able to calibrate the parameters solely based on the observation.

In this paper, a smart searching method was explored to find the best possible setup for the PPA using the biological data collected wirelessly using EMG. The smart search was achieved by a data-driven approach using multiple algorithms to investigate the speed and convergence of the methods. The most common way to search through the parameters is by doing an exhaustive search (ES), where all of the possible combinations of the parameters are explored and, based on the performance of the user in each combination, the best value is chosen. Even though looking through all of the possible combinations would result in an accurate tuning, this approach is time-consuming as there are thousands of combinations, and it would not serve as a practical replacement for the current methods. To overcome the time constraint of ES, we employ the genetic algorithm (GA), which is the most common heuristic optimization technique that has been used in various applications such as path planning, image processing, real-time systems, and so on [27]. GA has shown to be a very effective and an accurate approach for optimization. The genetic algorithm is a heuristic method that is inspired by the process of natural selection in order to generate the optimized solution. Genetic algorithms are often viewed as function optimizers, although the range of problems to which genetic algorithms have been applied is quite broad [28]. The implementation of GA starts with a population of random chromosomes and the population is evaluated using a fitness function. Based on the fitness, the members of the population are assigned a reproductive opportunity to find a better solution [28]. Using the goodness of the members, the parent chromosomes are selected for reproduction and, using a mutation technique, the next population gets generated and evaluated. This process is repeated until the user-defined stopping criteria is achieved. The stopping criteria was defined as the change in the best state in the current population and the best state in the previous iteration. However, as it uses a population-based optimization, which imitates the evolution theorem and updates the members of the population on every iteration, it might also require an unpractical number of trials to do the tuning. 

In this study, we also investigated the use of the Nelder–Mead (NM) simplex method to have a more optimal search and to find the best parameter combinations for the PPA calibration. The Nelder–Mead simplex algorithm is a very powerful local descent algorithm, making no use of the objective function derivatives. In NM, through a sequence of elementary geometric transformations (reflection, contraction, expansion, and multi-contraction), the initial simplex moves, expands, or contracts [29]. In addition, a modified NM method is introduced here by the addition of the Latin Hypercube Sampling (LHS) to limit the search area for improving the optimization and for finding the optimal parameters more accurately and faster. LSH uses a stratified sampling scheme to improve the coverage of the multi-dimensional input space and has been used in various computer models [30]. Using LHS, the conventional NM simplex method was modified to limit the searching area and to perform accurate tuning with fewer iterations.

## 3. Materials and Methods

In this section, the materials and methods used in this study are presented in four subsections. Firstly, the wireless body-area sensor network used to collect data is introduced. Secondly, the experimental in-lab protocol used to collect data, using the wireless EMG sensors, is explained. Thirdly, the signal processing method that was used to model the muscular activity from residual the limbs is presented. In the last section, various heuristic and numerical methods that were used to optimize the gait using EMG sensors are explained. 

### 3.1. Body-Area Sensor Network

This study employed a custom-made EMG body-area sensor network to collect the physiological signals from the residual limbs of transtibial amputees for muscular activity modeling and, subsequently, for the smart automatic tuning application on PPAs. The EMG signals were collected wirelessly using custom-made sensors, and the multi-channel EMG data was collected while the users operated a PPA. The bipolar derivation was used for the EMG recording; the mid-voltage at 0 V was used for the bias output to reduce the common-made effect. The self-designed prototype is a lightweight, low-power consumption, battery-powered, and wireless-enabled CyberSens for the EMG recording using an ADS-1292 chip. The ADS-1292 module is a low-cost, low-noise 24-bit analogue front-end biopotential measurement system, recently distributed by Texas Instruments (Dallas, TX, USA). Using an ADS-1292 module greatly reduced the cost of the CyberSens while maintaining the high-quality amplification. A bipolar power supply (+2.5 and −2.5 V) was used for ADS1292 to provide a large number of voltages. The ADS 1292 gain was set at 12 with the input voltage range from −0.2 to +0.2 V. A 32-bit Motion Processing Unit (MPU) (CC3200) with built-in Wi-Fi connectivity was employed in the CyberSens for on-board real-time signal processing and data transmission. This self-developed CyberSens provided a high-precision EMG signal with a less than 0.8 µV peak-to-peak noise. The custom-made CyberSens was designed to provide a seamless recording and the transmission of two channels of a 24-bit EMG signal, with the sampling rate up to 8000 Hz. The range of the wireless transmission can reach about 100 feet in an open space. Furthermore, an Inertial Measurement Unit (IMU) sensor was also embedded into the CyberSens. An MPU-9250 (Gyro, Accelerometer, and Magnetometer) Microelectromechanical systems (MEMS) motion tracking chip device (InvenSense, San Jose, CA, USA) was employed. This chip provides a user-programmable gyro full-scale range from ±250 to ±2000°/s and a user-programmable accelerometer full-scale range from ±2 g to ±16 g, which meets the requirement for studying human locomotion.

EMG is an experimental technique concerned with the development, recording, and analysis of myoelectric signals. Myoelectric signals are formed by physiological variations in the state of the muscle fiber membranes [31]. The EMG signal provides a window on the motor as well as on its controller [32]. The EMG data was collected from ten muscles in total, using surface Ag/AgCl electrodes from both limbs. The pre-gelled surface EMG electrodes (GS-27, provided by bio-medical.com) were used for the recording. The electrode placement was done by using the recommendations of the Surface EMG for Non-Invasive Assessment of Muscles (SENIAM) [33]. The following muscles were used in this study: Tibialis Anterior (TA), Gastrocnemius Lateralis (GL), Vastus Lateralis (VL), Rectus Femoris (RF), Biceps Femoris (BF), and Gluteus Medius (GM). The first two muscles (TA and GL) were collected from the intact limb, and the other four muscles were collected from both limbs. The data was collected wirelessly using the MATLAB platform and five in-lab designed sensors with the sampling frequency of 1000 Hz.

### 3.2. Experimental Protocol

**Figure 1:**
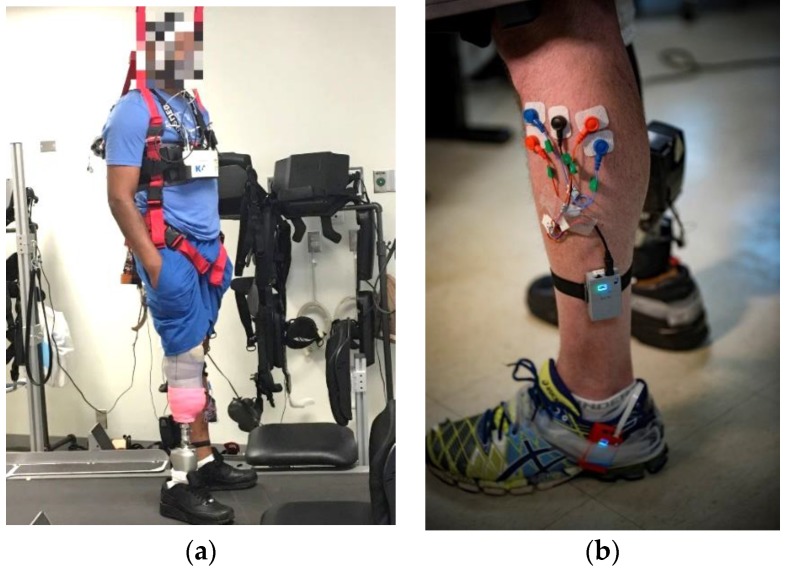
Data collection setup using a body weight supported treadmill system to prevent fall. (**a**) Amputee subject on the treadmill with a protective harness on (**b**) electrode placement on Tibialis Anterior (TA) and Gastrocnemius Lateralis (GL) muscles on the intact limb.

The subjects of this study consisted of three non-amputee subjects in a bent-knee (BK) configuration, and three TT amputees. The non-amputee subjects operated the leg in a bent knee position in order to simulate the amputee gait. Figure 1 shows a TT amputee and electrode placement for one wireless sensor with two channels of the EMG signal. The subjects operated the leg on a treadmill at a self-chosen comfortable walking speed. A harness was used to prevent the subjects from falling. Patient was fitted in a reasonable sized harness in a sitting position before mounting the treadmill. All straps were securely fastened to ensure appropriate support and free mobility during walking on the treadmill. A minimum support of 5% of subject’s body weight was provided during walking on the treadmill. The study was approved by the Institutional Board Review (IRB) in the Florida International University and the Hunter Holmes McGuire Veterans Administration Medical Center, and signed consent was obtained from the subjects prior to data collection. The Empower Ankle (Ottobock, Duderstadt, Germany), which was developed by Herr et al. [34], was used in the study. The main parameters for this ankle are the stiffness and power parameters. Each parameter varies from 0 to 100 percent, with a resolution of one percent. In this study, the power was limited to 50 percent in order to avoid over-powering and falling. The subjects were asked to walk on the treadmill for 10 steps, and various parameter combinations were recorded to be studied offline. The gold standard for metabolic energy expenditure is VO2, which shows the volume of the oxygen intake in milliliters per minute. In this study, the VO2 signal was collected from the TT subjects while they were operating the prosthetic leg. The VO2 system has a low frequency of one sample for every thirty seconds. The VO2 recordings did not show a meaningful statistical difference for 30, 90, 120, 150, and 180 s, and the *p*-value observed for all of the durations was more than 0.5. Therefore, a 10 steps (approximately 10 s) duration was chosen to be appropriate for this study. The VO2 system was not used for the rest of the study because of a lack of practical use, as it requires being recorded for at least three minutes in order to provide enough data. The stiffness parameter was varied from 0 to 100 percent, with steps of 10 percent, and the power was varied from 0 to 50 percent, with steps of five percent. In total, 121 various parameter combinations were collected in the recording. Later in the processing, the data was interpolated to include changes of one percent in the parameters, and 5151 parameters combinations were used in the optimization. Prior to the data collection, a CPO manually calibrated the PPA and the parameters were used to compare the results of the automatic tuning. The collected data was analyzed offline to find the best parameter combination, and various smart search methods were presented and tested to show the possibility of tuning the PPA automatically and faster. 

### 3.3. Modeling Muscular Activity Using EMG

EMG signals are non-stationary and noisy in nature, leading to complications in the comprehension of underlying information. In addition, the EMGs are highly susceptible to noise and are contaminated with a baseline drift, which depends on many factors such as the quality of the EMG amplifier, the environment noise, and the electrode movement [35]. The baseline drift lies in the lower frequencies and the main EMG information is limited to the bandwidth of 20–500 Hz [36]. To remove the baseline and high-frequency noise, the signals were filtered using a fourth-order Butterworth bandpass filter with cutoff frequencies of 20–500 Hz.

**Figure 2:**
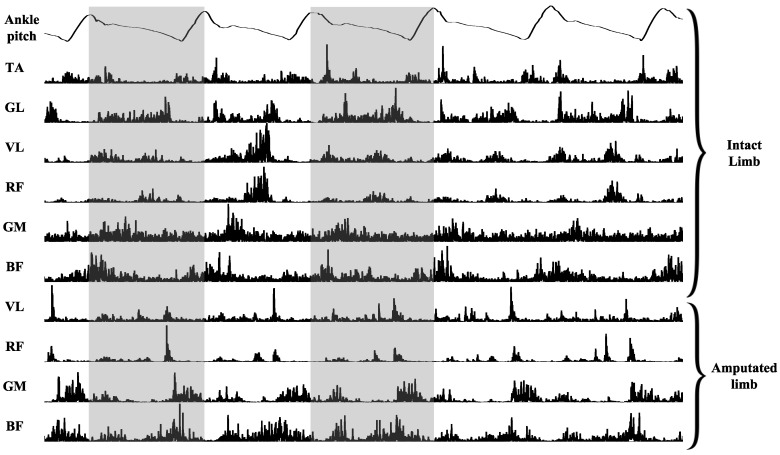
10 channels of EMG from lower limb of BK1 while operating PPA with zero percent power and zero percent stiffness for five steps. The pitch signals depicted on top was used to detect each step and it was used to partition EMG signals to each step. Two steps detected from the pitch signals are highlighted.

Furthermore, the gait phases were added to present an accurate phase-dependent model. The gait phases are detected using the IMU data that was incorporated in the sensor alongside EMG, and was recorded simultaneously. Human gait is a bipedal cycle, consisting of two phases—the stance phase and swing phase. The stance phase is the duration of contact between the ground and the feet, and swing phase is the duration when there is no foot contact with the ground [37]. The IMU signals collected on the ankle are used to estimate these phases. For this purpose, the accelerometer, gyroscope, and magnetometer data were collected using the nine-axis motion processing unit (MPU-9250), and using the real-time on-board processing pitch signal, were calculated and recorded. Using a threshold and peak detection algorithm, the timing for each step was detected and used to automatically partition the EMG signals into each step. Figure 2 illustrates the pitch signal on the top and two steps that have been detected are highlighted. 

As mentioned before, EMG signals are non-stationary and they need preprocessing to be able to extract information from them. Figure 2 shows 10 channels of synchronized filtered and rectified EMG, and the pitch signal with the detected extremums for step partitioning. To quantify the EMG signals, a simple feature extraction was implemented using the amplitude of the rectified signals. The feature selection was based on the fact that the EMG spectrum depends on the firing of the motor units, and it has been shown that it could be used to provide a sensitive measure for activity [38], and the amplitude of the signal shows the summation of the action potentials of the motor units firing around the electrode placement. It has been shown that the increase of the load on muscle increases the amplitude of the motor unit action potentials and, as a result, the amplitude of the EMG increases as well [39,40]. The changes in the EMG amplitude are correlated with the metabolic energy expenditure in the muscles. It was assumed that by focusing on the amplitude of the EMG signals, it is possible to estimate a measure to model the energy expenditure or load bearing on the residual limbs. For this purpose, the mean absolute value (*MAV*) feature was extracted as the following:

(1) $$\;MAV=\frac{1}{N}\overset{N}{\underset{i=1}{\sum }}\left|x\left[i\right]\right|\;$$

where *N* is the length of the EMG segment and x is the EMG signal.

The *MAV* feature is extracted from each of the ten muscles, and the average of the data collected from the first recording (0 Stiffness and 0 power) was used to normalize the features. The normalization coefficients are calculated as follows:

(2) $$\;n_{j}=\frac{1}{M}\overset{M}{\underset{k=1}{\sum }}{\left|MAV_{j}\left[k\right]\right|}_{\begin{array}{c} S=0\\ P=0 \end{array}}\;$$

where, *n* is the normalization factor for jth muscle, *M* is the number of steps, and *S* and *P* represent the stiffness and power parameters for PPA, respectively.

Previously, we have presented a method to use multi-channel EMG signals to model the amputee gait using a PPA, where different muscle groups were combined into a single measure using a weighted technique [26]. The weights for the muscles were calculated using the fast twitch fibre concentration [41]. Using the percentage of the fast twitch fibres in each muscle added the physical difference of the muscles to the model. The muscle size weights are also normalized with respect to the maximum to have values between 0 to 1 and the muscular activity model is calculated as follows:

(3) $$\;E\left(p,s\right)=\overset{N}{\underset{i=1}{\sum }}w_{i}\times \frac{MAV_{i}\left(p,s\right)}{n_{i}}\;\;$$

where $w_{i}$ is the normalized weight based on the concentration of the fast twitch fibers for the ith muscle and $MAV_{i}$ is the value of the phase dependent amplitude feature for the ith muscle, and $n_{i}$ is the normalization ration for ith muscle. 

The measure E(s,p) is a function of the parameters, which is the underlying model. But, because of the lack of data and non-stationary nature of EMG, it is unknown and it is calculated in a data-driven method here. 

### 3.4. Smart Search

The utilization of Equation (3) to identify the best parameters for each user would require doing an exhaustive search over all of the possible combinations for the stiffness and power parameters. As mentioned before, doing ES is accurate but unpractical because of the time constraint. It is also not efficient and the evaluation has a very high time/money/and so on, cost [42]. In addition, because of the subject variability and non-stationary nature of the EMG signals, the underlying model for energy is unknown. In this study, a data-driven smart search optimization was proposed to automatically tune the PPA and optimize Equation (3) without having to collect data for all of the possible combinations. Equation (3) is used as the objective function for the stiffness and power parameters, and the optimization was done as follows:

(4) $$\;\underset{p,s}{\mathrm{argmin}}E\left(p,s\right)\;\;s.t.\;0\leq p\leq 50\;\;0\leq s\leq 100$$

where *E* is the measure calculated using Equation (3), *p* is the power parameter, and *s* represents the stiffness parameter. As mentioned in Section 3.2, the upper limits for the parameters were determined to prevent over powering. 

**Figure 3:**
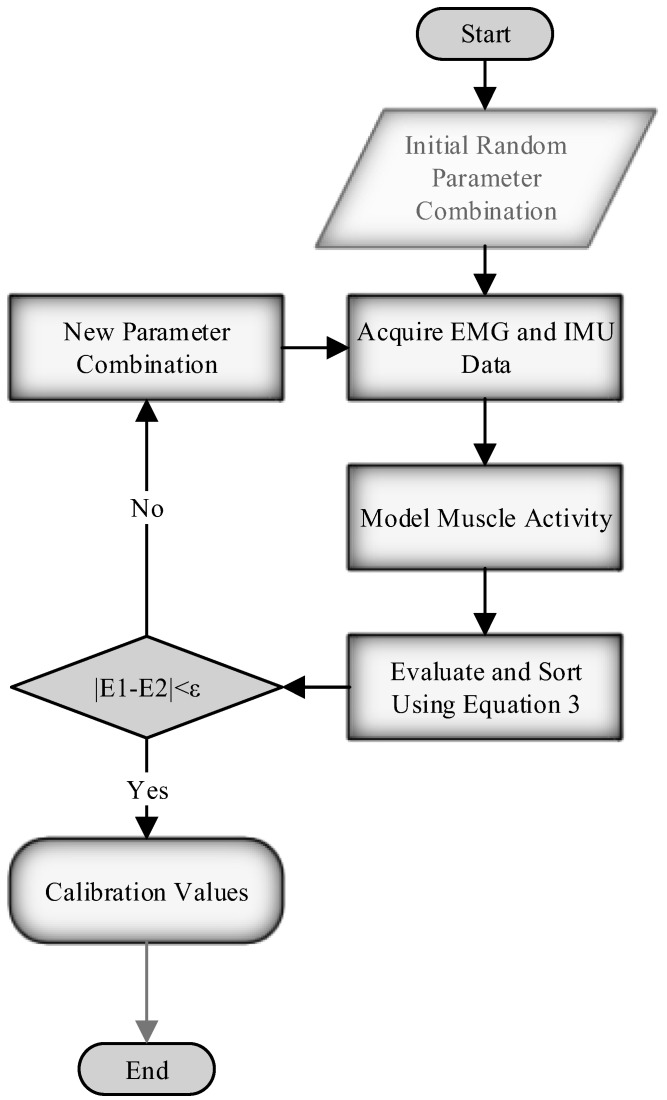
Flowchart of the proposed optimization in the study. *E*_1_ and *E*_2_ represent the smallest modelled muscle activity after they have been sorted and ε represents the user defined stopping criteria.

The study explored a more effective searching framework by employing heuristic and numerical methods to do the smart search to find the optimal tuning parameters. The data was collected offline from 121 combinations of stiffness and power, where the stiffness varied from 0 to 100 percent with a resolution of 10 percent, and the power varied from 0 to 50 percent with a resolution of five percent. The collection of data for 121 sessions was done for the sake of having the baseline grid for doing an exhaustive search and for implementing various optimization algorithms to compare the results. In addition, the data was interpolated after pre-processing to include a higher resolution for the parameters. The smallest change for each parameter in the systems is one percent, and the interpolation was done accordingly to have a one percent change in the power and stiffness parameters. The final grid contains 5151 possible combinations of the parameters. The interpolation was done in order to avoid the actual data collection, which would have required over 14 h of recording. To avoid doing ES over 5151 parameters, the alternative optimization algorithms of the genetic algorithm (GA) and the Nelder–Mead (NM) simplex methods were used in this study. In addition, a Latin Hypercube Sampling (LHS) approach was introduced to modify the NM method. Figure 3 shows the flowchart for the main idea of the optimization that has been proposed.

#### 3.4.1. Genetics Algorithm

The genetic algorithm is a heuristic search method inspired by Darwin’s evolution theorem. GA consists of four phases—initial population, fitness function, selection, and crossover and mutation. GA starts with an initial population, where each set is a possible solution, which in this case is the parameter combination. Each population is represented in the form of binary genes. Then, the population is evaluated by a fitness function. In this study, the fitness function is Equation (3). Based on the fitness value, the population is organized, and in the next phase, two individuals are selected for reproduction. After the parent selection, in the crossover phase, a crossover point in the chromosome is chosen to make offspring by exchanging the genes of the parents among themselves. To maintain diversity within the population, the mutation is done by randomly switching the values in a gene. This process is repeated until the stopping criteria is met or the maximum iterations are reached. In this study, the GA was modified to have integer values for parameters, and Equation (3) was used as the fitness function. The algorithm was explored using various numbers of populations to compare the speed and accuracy of the results.

#### 3.4.2. Nelder–Mead Simplex Method

The Nelder–Mead method is a numerical method that is commonly used to find the minimum or maximum of a multidimensional function, and it can be applied to nonlinear optimization problems where the derivatives may not be known. Its main strengths are that it requires no derivatives to be computed, and that it does not require the objective function to be smooth.

The NM method attempts to minimize a scalar-valued nonlinear function of the n variables using only the function values without any derivative information [43]. It is a direct data-driven searching method. NM uses geometrical shapes, where the corners are the vertices and the fitness of each vertex is used to expand or contract to converge to the optimal point. The worst vertices where E(p,s) has the biggest values is replaced with new vertices in each iteration, and a new triangle is formed. The vertices are updated in each iteration to form a new shape, where the value of the function gets smaller and smaller until the optimal point is found. In this study, various numbers of vertices were investigated to find the smallest number of vertices needed for this problem. The NM method iteratively generates a sequence of vertices to approximate an optimal point [44]. The method starts by randomly generating n+1 vertices [45]. At each iteration, the vertices are ordered according to the cost function values. In this study, the cost function, defined in Equation (3), was used to find power and stiffness values. Considering Equation (3), the ordered cost functions will be calculated as follows:

(5) $$\;E\left(p_{1},s_{1}\right)\leq E\left(p_{2},s_{2}\right)\leq \ldots \leq E\left(p_{n+1},s_{n+1}\right)\;\;$$

where, E denotes the cost function, and $p_{i}$ and $s_{i}$ are the randomly generated values for the stiffness and power parameters. As we are dealing with physical parameters, the random generation for the values were limited to suit the current problem. The power parameter was limited to 0 to 50 percent and the stiffness was limited to 0 to 100 percent. In addition, the randomly generated values are limited to integer values, as the resolution of the system cannot go under one percent. The algorithm uses four possible operations—reflection, expansion, contraction, and shrink—each being associated with a scalar parameter. Based on the value of the cost function, these possible operations are used to calculate the new point. After the initial n+1, the vertices are organized from best to worst, and the centroid for the n best vertices are calculated as follows:

(6) $$\;\bar{c}=\frac{1}{n}\overset{n}{\underset{i=1}{\sum }}c_{i}\;\;$$

where $c_{i}$ is a vector of the parameters as $\left[p_{i},c_{i}\right]$ in the ith vertices, *n* is the number of vertices, and $\bar{c}$ is the centroid that shows the coordinate of the centroid parameters. Using the centroid, a reflection point can be calculated as follows:

(7) $$\;c_{r}=\;floor\left(⎣\bar{c}+\alpha \left(\bar{c}-c_{n+1}\right)⎦\right)\;\;$$

where α is the reflection rate and the value is floored to have an integer value to fit the current problem, as the parameters have the constraint to obtain integer values. Following that, the function gets evaluated in the reflection point, and if $E_{1}\leq E_{r}\leq E_{n}$, then the n+1 vertices is replaced by $c_{r}$.

However, if $E_{r}<E_{1}$, then the expansion point needs to be calculated to cover the area with a smaller value that was missing in the previous iteration. The expansion point is calculated as follows:

(8) $$\;c_{e}=\;floor\left(⎣\bar{c}+\beta \left(c_{r}-\;\bar{c}\right)⎦\right)\;\;$$

where β is the expansion rate. The expansion point is evaluated and if $E_{e}<E_{r}$, $c_{n+1}$ is replaced by $c_{e}$. Otherwise, $c_{n+1}$ is replaced with $c_{r}$.

If the reflection point is $E_{n}\leq E_{r}<E_{n+1}$, then the algorithm needs to compute a contraction point outside and evaluate it as follows:

(9) $$\;c_{c}=\;floor\left(⎣\bar{c}+\gamma \left(c_{r}-\;\bar{c}\right)⎦\right)\;\;$$

where γ is the contraction rate. If $E_{c}\leq E_{r}$, then the contraction point replaces the n+1 point and, otherwise, the algorithm shrinks the data. Before going to the shrinking part, we need to consider the case where the reflection point is bigger than the n+1 point ($E_{r}\geq E_{n+1})$. In this case, we also need to contract the data inside using Equation (6). Evaluating the current contraction point, if it is smaller than hte n+1 point, it replaces that. Otherwise, the data needs to shrink as follows:

(10) $$\;c_{i}=\;floor\left(⎣c_{1}+\delta \left(c_{i}-c_{1}\right)⎦\right)\;\;$$

where δ is the shrink rate. Conventionally, the reflection, expansion, contraction, and shrink rates are user-defined, but in this study, we used the adaptive method presented by Gao et al. to implement the ND [44]. The adaptive parameters for NM method are calculated as the following:

(11) $$\alpha =1,\;\;\beta =1+\frac{2}{n},\;\;\gamma =0.75-\frac{1}{2\times n},\;\;\delta =1-\frac{1}{n}$$

The method was modified to fit our problem by limiting the parameters to integer values, which represent the one percent resolution, and the new point in each iteration was limited to the lower and upper bands of the parameters. The algorithm was also evaluated using various numbers of vertices to explore the smallest number required for tuning. The dimension of the vertices (*n*) was varied from 5 to 20, so as to evaluate the convergence accuracy and the number of necessary iterations. 

#### 3.4.3. Modified Nelder-Mean Complex with Latin Hypercube Sampling

If there are multiple local minima with small differences in the optimization problem, the NM might fail to converge to the global minima, and is stuck in the local minima. In addition, searching through a greater area might take more iterations until the target optimization value is achieved. To resolve this issue and improve the results, it is hypothesized that using a sampling method would improve the searching accuracy and find the minima in less iterations. For numerical problems, the Monte Carlo methods are usually more efficient to do a sampling from high dimensional probability distributions [46]. However, a great number of samples are typically required in the traditional Monte Carlo method, in order to achieve a good accuracy. Monte Carlo, which is a random sampling method, is the easiest method for sampling, but there is no assurance that a sample element will be generated from any particular subset of the sample space [47,48]. There are techniques to improve the accuracy by controlling the sample points. Latin Hypercube Sampling (LHS) is a widely used method to generate controlled random samples [49]. The basic idea of LHS is to divide the probability distributions into intervals of equal probabilities, and a sample is taken out of each interval [49,50,51]. In this study, two-dimensional LHS was used to obtain the sample points in order to determine the best search area. The two-dimension LHS was used with the assumption that the power and stiffness parameters are independent, their probability distributions are evenly partitioned into N regions, and one sample point is randomly selected from each region. By evaluating the samples, the area for the global minima is located. Using the located area, the searching method was limited to this area to keep the algorithm from being stuck in the local minima or from taking more iterations to find the global minima. In this study, the LHS sampling was combined with the NM method to improve the smart search result and to converge to the optimal setting faster. Using LHS, the NM method was restricted into the area specified by the sampling technique. Various initial points were investigated to evaluate the speed and accuracy of the tuning for PPA.

## 4. Results

**Figure 4:**
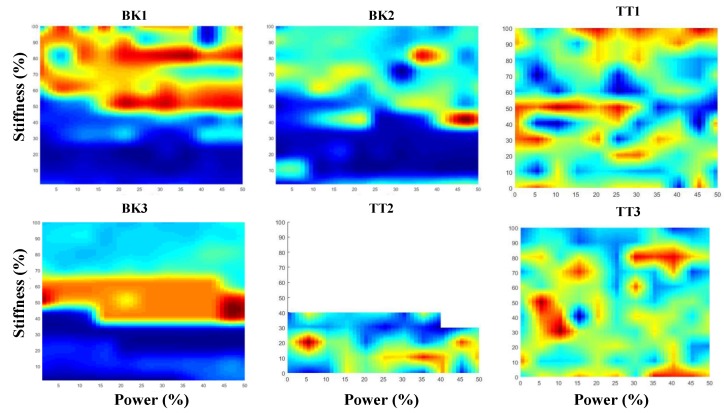
Grids of the modeled muscular activity using 10-channel electromyogram (EMG) sensor for various combinations of the stiffness and power parameters for three bent-knee and three trasfemoral amputee subjects.

Figure 4 shows the baseline grid for 5150 parameter combinations and the value of the estimated energy from EMG signals for six subjects (three amputees and three simulated amputees). The grid of the parameters shown here is collected using wireless sensors for 121 combinations of the parameters, and it was interpolated to have a resolution of one percent (smallest change in the parameters). The amplitude of the figures shows the modeled muscular activity using Equation (3). The subject variability is clearly observable on the grids, and each subject has a different measure in the obtained grid. Because of fatigue, TT2 was not able to finish the study and only half of the grid was obtained. The optimization was still implemented for the obtained half-grid for this subject, because the collected data included the CPO tuning value. Furthermore, the purpose of this study was for optimization, and it was only tested on the available grid.

The best parameters for each user are chosen to be the minimum for the modeled muscle activity in the grid, representative of the correct tuning value and it was used as the target value for the optimization evaluation. The collection of all of the possible parameter combinations for 10 steps (approximately 10 s) for 5150 parameter combinations would require the operation of the PPA for over fourteen hours, which is not practical, and the collection is not time-effective. 

Table 1 shows the results of the various methods presented in this study for each subject. It could be seen that GA has the best accuracy in finding the global minima. However, it requires more than 60 iterations and, in each iteration, there is a population that needs to be collected. The results are shown for a population of 15, which is more than 900 parameter combinations. In addition, the investigation of NM showed that it also converges to the target in a much shorter time (less than 40 iterations), but the variance is higher. The addition of boundaries using LHS reduced the variance and improved the search results, although requiring slightly more iterations. 

**Figure 5:**
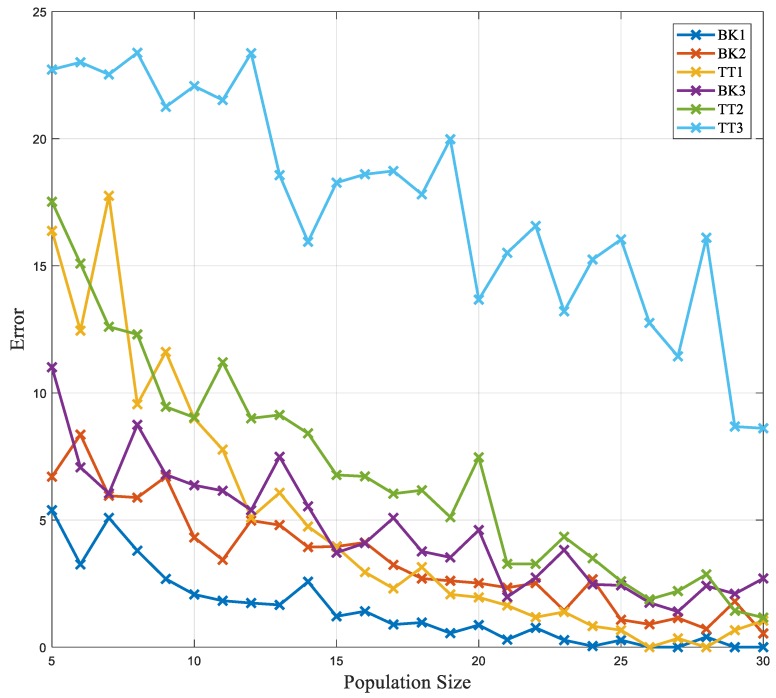
Error for smart search using the genetic algorithm (GA) method in various population sizes. The GA method was investigated using various initial population sizes to identify the smallest population size needed to converge to the global minima faster. The results are evaluated using an error measure, which is the distance of the result of the optimization from the target value.

The GA is implemented to do the search over the combinations in a heuristic way, so as to have a smarter alternative to the ES. Population sizes from 5 to 30 were investigated to explore the practicality of the search. Figure 5 shows the error bars for GA with various population sizes for each subject. The error is calculated as the Mahalanobis distance from the target point. With the increment in the population size, the error reduces, and the algorithm has a better convergence to the global minima. However, GA is population-based and every iteration requires an updated population and the collection of data for all of the members of the population. Table 1 shows the results of a search for all of the methods as well as the number of the iterations before convergence. 

**Figure 6:**
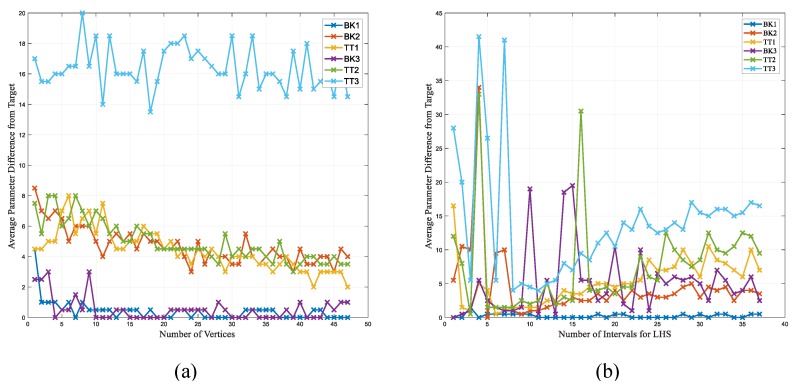
Error for smart search using Nelder–Mead (NM) and Latin Hypercube Sampling (LHS) methods in various vertices and random samples. (**a**) The distance from the target value for various simplexes. (**b**) The distance for the NM optimization using LHS with a various number of intervals to limit the search.

The NM method was explored using different vertices by sweeping *n* from 3 to 100. As the initialization is random, each *n* was repeated 1000 times and the mean error was calculated. Figure 6 shows the mean error for each of the vertices (*n*). The smallest number of *n* was chosen to be 10. Later, the NM method was explored by applying the search boundary determined by LHS. The LHS was explored with various starting points, and the starting points are swept from 4 to 30. Figure 6b shows the search results using various sampling points to bound NM. The best number of initial sampling was chosen to be five.

## 5. Discussion

In this study, a smart data-driven optimization method was used for automatic PPA tuning. A wireless EMG sensor system was used to collect physiological data from the muscles on the residual limbs of the subjects. Previously, some studies focused on automatically tuning prosthetic knees and ankles using mechanical sensors and did not consider the physiological aspect of the gait. The current study hypothesized that the use of physiological data could result in an accurate tuning. The reason for this hypothesis was based on the fact that the mechanical sensors collected information after the physical movement was made, while it is possible to use physiological data and observe the effect earlier. In addition, the physiological data is correlated to the energy expenditure, which can be used to optimize the performance of the PPA. For the physiological sensors, the EMG signals were collected from the residual limbs. The EMG signals are analyzed and combined using a weighted technique, where the weights reflected the size variability of the muscles based on the percentage of the fast twitch fibres.

The physiological data was processed and used to model the muscular activity. The estimated model was investigated to optimize the performance of the PPA by minimizing the muscle activity where the parameters were manipulated to tune the PPA. It was hypothesized that the normal gait has a smaller muscular activity and hence less energy expenditure, and the PPA should provide the smallest muscular burden on the subject to have the best performance. It has been shown that the muscular activity does increase in the amputee subjects when it is compared to the normal gait [52,53]. Therefore, by keeping the muscular activity low in the presence of prosthetic leg it is possible to improve the amputee gait. The current methods of tuning for the PPA are solely based on the observation of the CPO, and there is no tool to quantify the fitness of the leg. By introducing an EMG-based model, we presented an evidence-based alternative. The model considers the size difference of the various muscle groups by assigning a weight to the normalized amplitude feature of each muscle. It has been shown before that a similar method can be used to tune a PPA [26]. Table 1 shows the results of the CPO tuning and the result obtained from ES of the grid. The results showed comparable results using neural information and the neural information was used for smart optimization to avoid the burden of doing the ES. Figure 6 illustrates the average difference for the power and stiffness parameters, which is 4.1 and 11.1 percent. respectively. In a previous study, two independent CPOs tuned prosthetic limbs, and it was observed that it is possible to observe up to a five percent difference in the parameter setup, depending on the expert [26]. Hence, the 4.1 percent difference for the power parameter is chosen to be acceptable. On the other hand, the stiffness showed a bigger difference of 11.1 percent, which originated from the non-amputee users of BK2 and BK3. This inconsistency arises from using the simulated non-amputee subjects where the stiffness parameter does not affect the simulated gait. However, the purpose of this study was to minimize the muscle activity using a smart-optimization that would replace the time-consuming ES. Therefore, the use of non-amputee subjects have been determined to be suitable in order to test the convergence of the optimization, which later tests of the amputee subjects showed for the suitability of such a technique for the prosthetic tuning purpose. 

It is worth mentioning that the golden standard for the metabolic energy expenditure is VO2, and it was also collected from the subjects but the system has a low sampling frequency, requires at least three minutes of recording, and it would take at least an hour to collect just 20 parameter combinations [54,55]. This limitation will make subjects very tired and the fatigue might significantly affect the results for the parameter sets tested later. It has been shown that there are limitations in estimating the energy from the EMG signals, but using the proposed muscle activity model the study resulted in comparable parameters for the PPA [56,57]. This shows the possibility of using the muscular activity and tune the PPA by keeping the activity low. 

However, the best way to tune using the EMG-based method is to investigate the performance on all of the possible combinations for the stiffness and power parameters. Considering ten steps for each parameter combination, which is approximately ten seconds, would lead to a tuning session that would span over 14 h, which does not provide the time-efficiency for practicality. The focus of this study was to investigate the possibility of using adaptive data-driven optimization methods to achieve the parameter selection and to avoid ES. The GA and NM approaches were implemented and, later, a modification was added to the NM method using LHS to improve the search. GA resulted in the closest results to the target and smallest variance in the answers in comparison to others. GA was repeated 100 times; the results are reported for the average of the repetitions, and it is worth noting that over 90 percent of the results were exactly the target value. However, as expected, as this approach is population-based and requires having multiple combinations in every iteration, it requires many cases to be considered and, even though it improved the search with comparison to ES, it still is impractical for the tuning application. In this study, the NM simplex method was also proposed, where a geometrical simplex is manipulated by manipulating the shape using the fitness of the corners. A simplex, depending on the number of corners, would be a triangle, a foursquare, and so on. We investigated a triangular simplex of up to 100 simplex shapes in order to identify the smallest simplex to optimize the problem. Figure 6 showed the change in the mean difference from the target for 100 repetitions, and 10 simplexes were chosen as the best simplex for this purpose. The results of the NM method showed an average of less than 40 iterations to the set-up parameters, which indicates the possibility of doing automatic tuning in approximately six min on average. However, the variance of the results was more than five percent, which increases the inconsistency of the approach and might lead to over powering or under-powering of the PPA and the miscalculation for the stiffness. For example, the variance for the power of TT1 was 15, which is very large and it would not be practical to have this much inaccuracy. The small change in the muscle activity, which can be seen in Figure 4, is the reason for the big variance. The introduction of LHS to the NM method helped to limit the search to the area where the global minima was located, improved the results, and reduced the variance. However, the modified version converged to the target values in more iterations. It took 75 iterations at most, which is approximately 12 min, for the modified NM to tune the leg. The modified NM method using LHS resulted in a better accuracy in comparison to the NM, but it took longer to tune. However, the longer tuning time for the modified version was still short enough to be practical, and it can be used to replace the conventional subjective methods. In comparison to the GA, the modified NM was not as accurate, but it required significantly less time.

In this study, three non-amputee subjects were used in a bent-knee position to simulate the amputee gait for the PPA tuning. Even though it has been shown to be significantly different between the amputees and BK subjects [55], the results of the study were in accordance with the CPO tuning for the BK users. It is a rough estimation of the amputee gait, but the purpose for the study was reducing the muscular activity by tuning the PPA with the best parameters combinations.

Electromyographic signals are non-stationary and they change throughout the day. This might affect the sensitivity of the tuning. To avoid inconsistency, tuning needs to be tested on various days in different environments, in order to justify the accuracy of the proposed system. Furthermore, the presented algorithms are deterministic and might fail in noisy observations, which could be resolved by the incorporation of uncertainty, such as using Bayesian optimization [42]. However, the model used in this study is based on neural activity (EMG) and it is subject-dependent and non-stationary. This provides a limitation of having a mathematical model for the cost function. Because of this fact, it was decided to use NM, which does not require prior knowledge of the system, and the results showed the possibility of using such methods for PPA tuning.

The results of the study showed the feasibility of using physiological data to objectively tune a PPA automatically and accurately. In addition, the lack of a universal model for amputee gait because of the subject variability of the EMG signals was resolved by relying on the data itself. The NM collected data in a geometrical sense and, based on the fitness, the geometrical search area expanded or contracted to converge to the global minima, which are the best calibration values for the PPA parameters. Future work will focus on using approaches that deal with the uncertainty in the optimization, such as Bayesian optimization, and conducting an experiment on different days to validate that the approach would result in a similar tuning even with the changes. Finally, the study will focus on implementing a real-time system to be tested outside of a controlled environment.

## 6. Conclusions

The paper demonstrated the feasibility of using EMG signals from the residual limbs of amputees to model muscular activity for amputee gait. In addition, a modified NM optimization method was presented and its results show that it is possible to use the NM method as a smart automatic calibration tool for the setup of the PPA parameters. PPA calibration can be done objectively using the presented method, based on physiological evidence in a time-effective way.

## Figures and Tables

**Table 1:** Results of the electromyogram (EMG)-based smart search to tune powered prosthetic ankle (PPA). CPO—certified prosthetists and orthotists; LHS—Latin Hypercube Sampling.

Subject	CPO Tuning	Target ^1^	Genetic Algorithm		Nelder–Mead		Nelder–Mead with LHS	
			Iterations	Result	Iterations	Result	Iterations	Result
BK1	*P = 25* *S* = 25	*P* = 22 *S* = 21	990 ± 180	*P* = 22.3 ± 2 *S* = 20.7 ± 1.4	35 ± 9	*P* = 25 ± 7 *S* = 17 ± 5	49 ± 9	*P* = 24 ± 3 *S* = 19 ± 2
BK2	*P* = 20 *S* = 40	*P* = 23 *S* = 11	900 ± 105	*P* = 25.8 ± 7.2 *S* = 11.1 ± 1	28 ± 13	*P* = 31 ± 10 *S* = 14 ± 8	45 ± 8	*P* = 25 ± 6 *S* = 12 ± 2
TT1	*P* = 40 *S* = 32	*P* = 45 *S* = 26	870 ± 165	*P* = 44 ± 3 *S* = 25 ± 3	21 ± 8	*P* = 28 ± 15 *S* = 23 ± 9	57 ± 15	*P* = 43 ± 3 *S* = 25 ± 1
BK3	*P* = 40 *S* = 65	*P* = 46 *S* = 41	945 ± 150	*P* = 40 ± 12 *S* = 40 ± 6	10 ± 4	*P* = 27 ± 15 *S* = 51 ± 18	24 ± 13	*P* = 26 ± 0.7 *S* = 62 ± 1
TT2	*P* = 40 *S* = 40	*P* = 41 *S* = 31	870 ± 60	*P* = 39 ± 8 *S* = 29 ± 7	11 ± 8	*P* = 26 ± 12 *S* = 25 ± 11	13 ± 8	*P* = 40 ± 1 *S* = 31 ± 1
TT3	*P* = 23 *S* = 36	*P* = 16 *S* = 41	930 ± 150	*P* = 28 ± 15 *S* = 31 ± 11	9 ± 4	*P* = 28 ± 16 *S* = 32 ± 15	12 ± 2	*P* = 18 ± 6 *S* = 43 ± 8

^1^ P and S represent the power and stiffness parameters for PPA, respectively. BK stands for bent-knee and TT stands for transtibial.

## References

[1] Au S.K., Bonato P., Herr H. An EMG-position controlled system for an active ankle-foot prosthesis: An initial experimental study. Proceedings of the 9th International Conference on Rehabilitation Robotics, ICORR 2005.

[2] Fey N.P., Klute G.K., Neptune R.R. (2013). Altering prosthetic foot stiffness influences foot and muscle function during below-knee amputee walking: A modeling and simulation analysis. J. Biomech..

[3] Fey N.P., Klute G.K., Neptune R.R. (2012). Optimization of prosthetic foot stiffness to reduce metabolic cost and intact knee loading during below-knee amputee walking: A theoretical study. J. Biomech. Eng..

[4] Childers W.L., Kistenberg R.S., Gregor R.J. (2011). Pedaling asymmetries in cyclists with unilateral transtibial amputation: Effect of prosthetic foot stiffness. J. Appl. Biomech..

[5] Fey N.P., Klute G.K., Neptune R.R. (2011). The influence of energy storage and return foot stiffness on walking mechanics and muscle activity in below-knee amputees. Clin. Biomech..

[6] Robert P., Gailey R., Allen K., Castles J., Kucharik J., Roeder M. (2008). Review of secondary physical conditions associated with lower-limb amputation and long-term prosthesis use. J. Rehabil. Res. Dev..

[7] Sewell P., Noroozi S., Vinney J., Andrews S. (2000). Developments in the trans-tibial prosthetic socket fitting process: A review of past and present research. Prosthet. Orthot. Int..

[8] Hanspal R., Fisher K., Nieveen R. (2003). Prosthetic socket fit comfort score. Disabil. Rehabil..

[9] Popovic D., Tomovic R., Tepavac D., Schwirtlich L. (1991). Control aspects of active above-knee prosthesis. Int. J. Man-Mach. Stud..

[10] Bédard S., Roy P.-O. (2008). Actuated Leg Prosthesis for Above-Knee Amputees. Google Patent.

[11] Herr H.M., Grabowski A.M. (2012). Bionic ankle-foot prosthesis normalizes walking gait for persons with leg amputation. Proc. R. Soc. B.

[12] Holmberg W.S.U. (2006). An autonomous control system for a prosthetic foot ankle. IFAC Proc. Vol..

[13] Liu M., Datseris P., Huang H.H. (2012). A prototype for smart prosthetic legs-analysis and mechanical design. Advanced Materials Research.

[14] Sup F., Bohara A., Goldfarb M. (2008). Design and control of a powered transfemoral prosthesis. Int. J. Robot. Res..

[15] Au S.K., Herr H., Weber J., Martinez-Villalpando E.C. Powered ankle-foot prosthesis for the improvement of amputee ambulation. Proceedings of the 29th Annual International Conference of the IEEE Engineering in Medicine and Biology Society.

[16] Koniuk W. (2002). Self-Adjusting Prosthetic Ankle Apparatus. Google Patents.

[17] Li C., Tokuda M., Furusho J., Koyanagi K., Morimoto S., Hashimoto Y., Nakagawa A., Akazawa Y. Research and development of the intelligently-controlled prosthetic ankle joint. Proceedings of the 2006 IEEE International Conference on Mechatronics and Automation.

[18] Williams R.J., Hansen A.H., Gard S.A. (2009). Prosthetic ankle-foot mechanism capable of automatic adaptation to the walking surface. J. Biomech. Eng..

[19] Wang D., Liu M., Zhang F., Huang H. Design of an expert system to automatically calibrate impedance control for powered knee prostheses. Proceedings of the 2013 IEEE International Conference on Rehabilitation Robotics (ICORR).

[20] Huang H., Crouch D.L., Liu M., Sawicki G.S., Wang D. (2016). A cyber expert system for auto-tuning powered prosthesis impedance control parameters. Ann. Biomed. Eng..

[21] Wen Y., Brandt A., Liu M., Huang H.H., Si J. Comparing parallel and sequential control parameter tuning for a powered knee prosthesis. Proceedings of the 2017 IEEE International Conference on Systems, Man, and Cybernetics (SMC).

[22] Cain S.M., Gordon K.E., Ferris D.P. (2007). Locomotor adaptation to a powered ankle-foot orthosis depends on control method. J. Neuroeng. Rehabil..

[23] Sawicki G.S., Ferris D.P. (2008). Mechanics and energetics of level walking with powered ankle exoskeletons. J. Exp. Biol..

[24] Ferris D.P., Gordon K.E., Sawicki G.S., Peethambaran A. (2006). An improved powered ankle–foot orthosis using proportional myoelectric control. Gait Posture.

[25] Atri R., Marquez J.S., Murphy D., Gorgey A., Fei D., Fox J., Burkhardt B., Lovegreen W., Bai O. Investigation of muscle activity during loaded human gait using signal processing of multi-channel surface EMG and IMU. Proceedings of the Signal Processing in Medicine and Biology Symposium (SPMB).

[26] Atri R., Sebastian Marquez J., Murphy D., Gorgey A., Fei D., Fox J., Lovegreen W., Bai O. EMG-based energy expenditure optimization for active prosthetic leg tuning. Proceedings of the 2017 39th Annual International Conference on Engineering in Medicine and Biology Society (EMBC).

[27] Kumar M., Husian M., Upreti N., Gupta D. (2010). Genetic algorithm: Review and application. Int. J. Inf. Technol. Knowl. Manag..

[28] Whitley D. (1994). A genetic algorithm tutorial. Stat. Comput..

[29] Chelouah R., Siarry P. (2003). Genetic and Nelder–Mead algorithms hybridized for a more accurate global optimization of continuous multiminima functions. Eur. J. Operat. Res..

[30] Iman RL Latin Hypercube Sampling. https://onlinelibrary.wiley.com/doi/abs/10.1002/9781118445112.stat03803.

[31] Konrad P. (2005). A Practical Introduction to Kinesiological Electromyography.

[32] Merletti R., Parker P.A. (2004). Electromyography: Physiology, Engineering, and Non-Invasive Applications.

[33] Hermens H.J., Freriks B., Merletti R., Rau G. (1999). European recommendations for surface electromyography. Roessingh Res. Dev..

[34] Herr H.M., Han Z., Barnhart C.E., Casler R. (2015). Prosthetic, Orthotic or Exoskeleton Device. Google Patents.

[35] Konrad P. (2005). The ABC of EMG: A Practical Introduction to Kinesiological Electromyography.

[36] De Luca C.J. (1997). The use of surface electromyography in biomechanics. J. Appl. Biomech..

[37] Iosa M., Fusco A., Marchetti F., Morone G., Caltagirone C., Paolucci S., Peppe A. (2013). The golden ratio of gait harmony: Repetitive proportions of repetitive gait phases. BioMed Res. Int..

[38] Moritani T., Muro M. (1987). Motor unit activity and surface electromyogram power spectrum during increasing force of contraction. Eur. J. Appl. Physiol. Occup. Physiol..

[39] Yana K., Mizuta H., Kajiyama R. Surface electromyogram recruitment analysis using higher order spectrum. Proceedings of the IEEE 17th Annual Conference on Engineering in Medicine and Biology Society.

[40] Reaz M.B.I., Hussain M., Mohd-Yasin F. (2006). Techniques of EMG signal analysis: Detection, processing, classification and applications. Biol. Proced. Online.

[41] Umberger B.R., Gerritsen K.G., Martin P.E. (2003). A model of human muscle energy expenditure. Comput. Methods Biomech. Biomed. Eng..

[42] Brochu E., Cora V.M., De Freitas N. (2010). A tutorial on Bayesian optimization of expensive cost functions, with application to active user modeling and hierarchical reinforcement learning. arXiv.

[43] Lagarias J.C., Reeds J.A., Wright M.H., Wright P.E. (1998). Convergence properties of the Nelder-Mead simplex method in low dimensions. SIAM J. Optim..

[44] Gao F., Han L. (2012). Implementing the Nelder-Mead simplex algorithm with adaptive parameters. Comput. Optim. Appl..

[45] Singer S., Nelder J. (2009). Nelder-mead algorithm. Scholarpedia.

[46] Hastings W.K. (1970). Monte Carlo sampling methods using Markov chains and their applications. Biometrika.

[47] Helton J.C., Davis F.J. (2003). Latin hypercube sampling and the propagation of uncertainty in analyses of complex systems. Reliabil. Eng. Syst. Saf..

[48] Ye T., Kalyanaraman S. (2003). A recursive random search algorithm for large-scale network parameter configuration. ACM Sigmetrics Perform. Eval. Rev..

[49] McKay M.D., Beckman R.J., Conover W.J. (2000). A comparison of three methods for selecting values of input variables in the analysis of output from a computer code. Technometrics.

[50] Olsson A.M., Sandberg G.E. (2002). Latin hypercube sampling for stochastic finite element analysis. J. Eng. Mech..

[51] Owen A.B. (1994). Controlling correlations in Latin hypercube samples. J. Am. Stat. Assoc..

[52] Wentink E.C., Prinsen E.C., Rietman J.S., Veltink P.H. (2013). Comparison of muscle activity patterns of transfemoral amputees and control subjects during walking. J. Neuroeng. Rehabil..

[53] Huang S., Ferris D.P. (2012). Muscle activation patterns during walking from transtibial amputees recorded within the residual limb-prosthetic interface. J. Neuroeng. Rehabil..

[54] Levine J.A. (2005). Measurement of energy expenditure. Publ. Health Nutr..

[55] Waters R.L., Mulroy S. (1999). The energy expenditure of normal and pathologic gait. Gait Posture.

[56] Seliger V., Dolejš L., Karas V. (1980). A dynamometric comparison of maximum eccentric, concentric, and isometric contractions using EMG and energy expenditure measurements. Eur. J. Appl. Physiol. Occup. Physiol..

[57] KyrÖlÄinen H., Belli A., Komi P.V. (2001). Biomechanical factors affecting running economy. Med. Sci. Sports Exerc..

